# Unraveling reward processing in schizophrenia and bipolar disorder: a multilevel examination of the positive valence system

**DOI:** 10.1017/S0033291726103444

**Published:** 2026-04-27

**Authors:** Monique van der Weijden-Germann, Neeltje E.M. van Haren, Marisha N. Meijer, Marit I. Broer, Annabel Vreeker, Laura J. van den Brink-Steinmann, Sofie Paludanus, Roel Ophoff, Robert M. Bilder, Marjolein E.A. Barendse

**Affiliations:** 1 Child and Adolescent Psychiatry/Psychology, Erasmus University Medical Centre – Sophia, Rotterdam, Netherlands; 2 Department of Psychology, Education and Child studies, Erasmus University Rotterdam, Rotterdam, The Netherlands; 3 Brain Research and Innovation Centre, Ministry of Defence, Utrecht, the Netherlands; 4 Department of Psychiatry, University Medical Centre Utrecht Brain Centre, Utrecht, the Netherlands; 5 Force Health Protection, Netherlands Armed Forces, Ministry of Defence, Doorn, the Netherlands; 6 Semel Institute for Neuroscience and Human Behavior, University of California Los Angeles, Los Angeles, CA, USA

**Keywords:** familial high-risk, mood, psychosis, reward processing, task performance

## Abstract

**Background:**

The positive valence systems (PVS) domain, a key focus of the Research Domains Criteria framework, divides reward-related processes into three constructs: reward responsiveness, reward learning, and reward valuation. Difficulties with several of these reward constructs have been reported in people with mood-psychosis spectrum disorders. This study aims to examine how performance on tasks corresponding to these three constructs covaries, and how performance relates to mood and psychotic symptoms in adults with mood-psychosis spectrum disorders, those at familial risk, and controls.

**Methods:**

Data from two studies (*N* = 278 and *N* = 332) were analyzed, which both included people with a psychotic disorder or bipolar disorder (patients), their first-degree relatives (FDRs), and controls. PVS constructs were measured using the Multi-Armed Bandit Task, Effort-Expenditure for Rewards Task, and Monetary Incentive Delay Task. Depression, mania, and psychosis symptoms were measured with self-report and interview instruments. Confirmatory factor analysis was used to examine covariation, and path analysis to test associations with symptoms.

**Results:**

The three reward constructs showed weak (nonsignificant) covariance in all groups. There were a few impairments in reward-related performance in patients or FDRs, none that survived multiple-comparison correction. There were no associations between symptoms and performance on the PVS constructs after multiple comparisons correction.

**Conclusions:**

The findings showed no evidence that performance on any of the three PVS constructs could constitute an endophenotype of mood-psychosis spectrum disorders. We recommend future research examining the contribution of specific cognitive skills to reward-related behavior, and to sources of heterogeneity in reward functioning within the patient group.

## Introduction

Reward processing refers to the psychological, behavioral, and neural mechanisms by which individuals detect, evaluate, anticipate, and respond to experiences of reward and pleasure. To improve characterization of this multidimensional construct, the Research Domain Criteria framework was developed (NIMH, 2008). This includes the ‘*Positive Valence Systems (PVS)*’ domain, which is divided into the constructs of reward responsiveness, reward learning and reward valuation (NIMH, 2023). Reward responsiveness refers to the capacity to respond to, enjoy, and seek out rewarding stimuli, encompassing both initial reactions and sustained engagement with positive experiences. Reward learning relates to the ability to modify behavior based on experience with rewards, involving learning associations between stimuli, actions, and outcomes. Finally, reward valuation refers to processes by which an individual assesses the value of a potential reward based on its probability, cost/effort, and context (NIMH, 2023). Reward processing plays an important role in shaping behavior and motivation, and impairments could be expressed behaviorally as anhedonia, reduced motivation for social events, or impaired self-care (Alloy & Nusslock, 2019).

Such behaviors are often reported across disorders in the mood-psychosis spectrum (Der-Avakian & Markou, 2012; Strauss & Gold, 2012), such as schizophrenia and bipolar disorder (BD). These disorders demonstrate considerable overlap in both genetic predisposition and phenotypic expression (Förstner et al., 2023).

Several studies report different degrees of functioning across the PVS construct of reward responsiveness in individuals with psychotic disorders, BD, and major depressive disorder (MDD). That is, the immediate response to reward in an experimental setting appears to be intact in individuals with psychotic disorders (e.g. schizophrenia and schizoaffective disorder) or BDs (Johnson et al., 2019; Strauss, Visser, Lee, & Gold, 2017; Yan et al., 2012). Self-reported anticipatory pleasure was found to be intact in BDs (Tso, Grove, & Taylor, 2014); however, impaired in individuals with a psychotic disorder or MDD (Frost Visser et al., 2020; Hallford et al., 2019). The same is true for neural responses in the striatum during reward anticipation (Long et al., 2022; Yang et al., 2022; Zeng et al., 2022).

Impairments in reward learning, specifically in identifying the most rewarding option, have been documented in individuals with schizophrenia (Koch et al., 2010; Waltz, Frank, Robinson, & Gold, 2007; Waltz, Frank, Wiecki, & Gold, 2011; Yılmaz, Simsek, & Gonul, 2012), BD (Brambilla et al., 2013), and MDD (Adida et al., 2011; Dezfouli et al., 2019; Halahakoon et al., 2020). However, these impairments do not appear to extend to punishment learning (Adida et al., 2011; Strauss et al., 2011; Waltz et al., 2007). Optimal reward learning requires the ability to discern the overall reward patterns associated with each option without an overreliance on recent successes or failures. In BD, difficulties might be driven by high sensitivity to (recent) wins (Brambilla et al., 2013). In schizophrenia, reward learning performance has been inversely associated with negative but not with psychotic symptoms (Strauss et al., 2011; Waltz et al., 2007; Waltz et al., 2011; Yılmaz et al., 2012). Depressive symptoms in BD have been linked to poorer reward learning, whereas mania symptoms have not (Adida et al., 2011; Pouchon et al., 2023). Despite these findings, there remains limited research exploring the associations of psychotic and manic symptoms with reward learning, particularly from a transdiagnostic perspective.

Reward valuation includes weighing, for example, effort and probability in decisions to pursue a reward, and therefore it is less obvious what constitutes an impairment. Generally, people with psychotic disorders take less effort for reward in the most rewarding conditions (Barch, Treadway, & Schoen, 2014; Gold et al., 2013; Yang et al., 2021; Zou et al., 2020). This is stronger for those with many negative symptoms (Yang et al., 2021; Zou et al., 2020), and similar findings have been reported in people with depression and those who score high on depressive symptoms (Halahakoon et al., 2020). For BD, findings on reward valuation have been mixed (Hayden et al., 2008; Hershenberg et al., 2016; Yang et al., 2021; Isıklı et al., 2024; Moran, Prevost, Culbreth & Barch, 2024).

Taken together, impairments seem to exist in all three domains of the PVS in people with psychotic disorders, and, though sometimes less consistently, BD. The strongest impairments might exist in those with more severe negative or depressive symptoms. In line with this, a trans-diagnostic study employing a sample of individuals along a severe mental illness-spectrum reported that global reward processing deficits (i.e. across the three PVS domains) are associated with negative symptoms, while a selective weakened reward response is not (Luther, Jarvis, Spilka, & Strauss, 2024).

Though domains of reward processing have been suggested as potential endophenotypes of BD or SCZ, the findings in first-degree relatives (FDRs) of individuals with mood-psychosis spectrum disorders have been mixed (Bora et al., 2024; Hanssen, Krabbendam, Robberegt, & Fett, 2020; Linke et al., 2020). Several studies found no behavioral differences between people with psychosis, their FDRs, and healthy individuals during reward anticipation (Mucci et al., 2015; Millman et al., 2020). A study found equal reward learning rates in BD relatives and controls (Linke et al., 2020), while relatives of people with schizophrenia were found to have difficulty with reward learning (Hanssen et al., 2020). Young adults with clinical high risk for psychosis or BD showed lower effort for reward in highly rewarding conditions (Bora et al., 2024), but as far as we know, this has not been investigated in familial high-risk groups. Compared to healthy individuals, first-degree relatives of people with BD showed decreased prediction error signals during a reward task (Macoveanu et al., 2020). It is important to note that the extent of literature on relatives is much more limited than that on patients, and most studies had small samples, so additional studies with larger sample sizes are needed to establish whether functioning on any of the three PVS domains constitutes an endophenotype. The identification of such endophenotypes could aid in bridging the gap between genetic risk and clinical manifestation, and offer potential targets for early intervention (Gottesman & Gould, 2005).

The majority of research studied each of the PVS components in relative isolation, and limited data are available on how these three components covary on a behavioral level. However, studies have assessed the relationship between two reward concepts. For instance, Nguyen et al. (2022) and Duffy et al. (2024) found no correlation between effort expenditure and reward learning in a healthy population. Another study reported no significant association between reward learning and self-reported reward responsiveness (Sailer, Wurm, & Pfabigan, 2024). Geaney et al. (2015) revealed a significant but weak positive correlation between reward anticipation and effort expenditure at low probabilities. These weak associations between the reward constructs are surprising, as research has demonstrated that the components involve overlapping neural and genetic pathways (Hess et al., 2016). An important point for future research is to determine whether these results hold true in patient populations.

Although recent findings on reward-processing components have advanced our understanding of shared and distinct features of disorders across the mood-psychosis spectrum, research to date has primarily focused on individual reward-processing components in isolation. Consequently, little is known about how these components covary at a behavioral level and whether such covariation is different in those with a mood-psychosis spectrum disorder compared to the general population. Understanding the interplay between PVS components is important to better understand the origins of deficits, if deficits in multiple domains exist. In addition, examining PVS performance in FDRs of people with mood-psychosis spectrum disorder aids in bridging the gap between genetic risk and clinical manifestation. Reward-processing deficits in relatives would indicate familial liability as an underlying mechanism, whereas the absence of those deficits but associations with current symptoms would suggest that deficits are state-dependent. Therefore, the current preregistered (https://doi.org/10.17605/OSF.IO/WQBNE) study addressed the following aims.

First, we aimed to determine how PVS components covary on a behavioral level in the overall cohort of individuals with a mood-psychosis spectrum disorder, their FDRs, and controls (RQ1). Based on the above outlined study results demonstrating no or weak correlations between two PVS components, we hypothesized that we would similarly find weak or no covariation between the three PVS components.

Second, we examined to which extent this covariation differs between individuals with a mood-psychosis spectrum disorder, FDRs, and controls (RQ2). Based on these findings, we expect to find considerable variability in reward processing in patients. Therefore, we hypothesized that the covariation between PVS components would be less pronounced in patient groups as compared to the relatives and the control group.

Third, we examined to which extent individuals with a mood-psychosis spectrum disorder, their FDRs, and controls differ in performance on the PVS components (RQ3). We expected that individuals with psychotic or BDs would show worse performance on tasks measuring reward anticipation, reward learning, and willingness to expend effort for reward compared to controls. FDRs are furthermore expected to exhibit intermediate profiles, reflecting their familial liability and subclinical phenotypic traits.

Fourth, we analyzed how current depression, mania, and psychosis symptoms are related to performance on the PVS components (RQ4). We hypothesize that deficits in performance on the PVS components are related to negative and depressive symptoms, but not mania or psychosis symptoms.

## Materials and methods

### Study design and procedure

Data from the current investigation stem from two substudies, which both aimed to investigate the PVS in people with BD or a psychotic disorder, their FDRs, and controls. First, we set up a cross-sectional family design neuroimaging study (from hereon in-lab study). at the Erasmus Medical Center (Erasmus MC) in Rotterdam, The Netherlands. Participation in this study entailed one visit with a duration of ~5.5 h, including diagnostic interviewing, a magnetic resonance imaging (MRI) scan, three computer tasks, the collection of blood samples, and assessments of blood pressure, height, and weight. Finally, participants completed self-report questionnaires on-site or at home. Second, an observational family design online study was conducted (from hereon online-study; Broer et al., 2025, preprint). Eligible participants received a link to the information letter and informed consent form. They completed questionnaires and the three tasks that were also used in the in-lab study, on a computer at home, at any time convenient to them.

Both studies were approved by the local medical ethical committee of the Erasmus MC, and all procedures were conducted in accordance with the Declaration of Helsinki (64th WMA General Assembly; October 2013). Written informed consent was obtained from all participants before participation. Data were collected between January 2021 and August 2024. The hypotheses and methods were preregistered at: https://doi.org/10.17605/OSF.IO/WQBNE.

### Participants and recruitment

For both studies, recruitment of participants was accomplished by directly contacting participants from the Dutch Bipolar Cohort (Snijders et al., 2020; Vreeker et al., 2016) and Genetic Risk and Outcome of Psychosis study (Korver et al., 2012). For the in-lab study, additional recruitment took place via patient organizations and psychiatric clinics in or near Rotterdam, The Netherlands. Participants who declined participation in the in-lab study or did not meet its inclusion criteria were also invited to take part in the online-study. The existing cohorts included participants aged ≥18 years. For the in-lab study, 278 participants signed informed consent; for the online-study, 332 participants signed informed consent.

For both studies, participants needed to demonstrate understanding of the purpose, procedures, risks, and benefits, and payment procedures of the present investigation. For the in-lab study, individuals were not eligible for participation if they presented MRI-related contraindications (i.e. claustrophobia, non-removable ferromagnetic objects, pacemaker, or other stimulatory devices), if they had a history of closed head injury, neurological illness, and endocrine dysfunction, or if they demonstrated neurological abnormalities and/or structural brain abnormalities that interfere with present assessments, based on neurodiagnostic evaluation. For the online-study, individuals had to provide informed consent through an online portal and be able to complete the assessments on a computer (not tablet or mobile device).

Participants were included in the patient group if they had BD type 1, type 2, or not otherwise specified (NOS), schizophrenia, schizoaffective disorder, schizophreniform disorder, delusional disorder, brief psychotic disorder, or psychotic disorder NOS. Relatives were required to have a first-degree family member with one of these diagnoses, but did not have these diagnoses themselves. Individuals from the healthy control group were eligible if they themselves and their first-degree family members were free of the diagnoses mentioned for the patient group. Relatives and controls were still eligible for participation in the presence of other psychopathologies. For the online-study, diagnostic status was based on information from the prior cohorts or from the clinic. For the in-lab study, diagnoses were confirmed with diagnostic interviews conducted by trained researchers (using the Comprehensive Assessment of Symptoms and History (Andreasen, Flaum, & Arndt, 1992) for participants with a bipolar or psychotic disorder based on information from the prior cohorts or the clinic, using the Mini-International Neuropsychiatric Interview for other participants (Sheehan et al., 1998). The Diagnostic and Statistical Manual of Mental Disorders, Fifth Edition criteria were followed, and decisions were made based on consensus between two trained researchers. For the FDR group, the family member with mental illness could participate themselves as well, but if not, their diagnostic status was confirmed as part of the prior cohorts we recruited from.

### Tasks

The order of the computer tasks was counterbalanced. Each task was preceded by instructions on screen. The participants were told that their performance on the computer tasks influenced the amount of reward received, but in reality, they received a fixed amount at the end of all assessments. This was explained to them during a debriefing (in-person for in-lab study, as written text for online-study). Detailed descriptions of each task can be found in the Supplementary Materials. The code and stimuli of the tasks are freely available at DOI: 10.17605/OSF.IO/2WY7U.

The monetary incentive delay (MID) task was intended to measure anticipation of reward. Outcome parameters extracted from this task entail the difference in average accuracy and reaction time between reward (combining small and large rewards) and neutral trials.

The Effort Expenditure for Reward Task (EEfRT) (Treadway et al., 2009) evaluates how participants assess effort versus reward. Outcome parameters retrieved from this task entail the overall percentage of hard-task choices, the linear slope in hard-task choices as a factor of hard-task reward magnitude (separately at both reward probabilities), and the difference in percentages of hard-task choices between the two probabilities. In calculating an individual’s slope in hard-task choices, we covary with trial number to remove any effects of fatigue.

Participants played two versions of the three-armed Bandit task: (1) the coin Bandit task, and (2) the social Bandit task, measuring financial and social reward learning, respectively. Outcome measures were calculated separately for each version and consisted of the total score, the win-stay rate, the lose-shift rate, and the learning rate. The win-stay rate is defined as the percentage of times a participant chooses the same arm after being rewarded by that arm in the previous trial, averaged across all rounds. The lose-shift rate is defined as the percentage of times a participant chooses a different arm after not being rewarded by an arm in the previous trial, averaged across all rounds. The learning rate is the linear slope of the likelihood of choosing the best arm as a factor of the trial number. This is calculated for the first half of all rounds, as most learning takes place then, and the slope is likely to get nonlinear in the second half of the runs. The best arm is the one that has the highest reward rate in that round.

### Symptom measures

For the in-lab study, we used the total score of the Altman Self-Rating Mania (ASRM) scale (Altman, Hedeker, Peterson, & Davis, 1997) as a measure of mania symptoms, the total score of the Inventory of Depressive Symptomatology (IDS) self-report (Rush et al., 1996 28) for depressive symptoms. The ASRM and IDS were self-report questionnaires. The positive and negative symptom scores were assessed using the Positive and Negative Syndrome Scale (PANSS) interview (Kay, Fiszbein, & Opler, 1987). Relatives and controls completed only the IDS, as the ASRM and PANSS are developed for and validated in people with a bipolar or psychotic disorder specifically.

For the online-study, symptom assessments were self-reported. We used the total score of the ASRM for mania symptoms, the total score on the 8-item Patient Reported Outcomes Measurement Information System (PROMIS, Pilkonis et al., 2011) depression scale (Cella, Gershon, Bass, & Rothrock, 2015) for depressive symptoms, and the total score of the psychosis subscale of the BASIS-24 (Cameron et al., 2007) for psychotic symptoms. There was no index of negative symptoms in the online-study.

### Analysis plan

The steps to answer all research questions are outlined in detail in the Supplementary Materials and summarized below; we executed these steps separately in the in-lab study and online-study. We used R’s lavaan package. First, we performed assumption checks for normality, outliers, multicollinearity, and homoscedasticity, and conducted corrections if necessary. Second, we used linear mixed-effect modeling to test group differences in all observed task outcome variables (as defined in Section ‘Tasks’ and listed on the left in Supplementary Figure S1), corrected for age, sex, and years of education, and with a random intercept by family ID (first part of RQ3). The multiple comparisons corrected threshold was *ɑ* = .005. Third, to examine the covariation among the PVS components, multilevel confirmatory factor analysis (CFA) was used (RQ1). The model from Supplementary Figure S1 with the three covariates (but without the ‘symptoms’ variable) was used as the basis and modified, for example, by correlating errors, to improve fit. Fourth, we used multigroup CFA on the model from the previous step to compare the factor covariances between groups (RQ2). We extracted factor scores from the three factors using lavPredict and applied analysis of covariance (ANCOVA) to compare these scores between groups (second part of answer to RQ3). The corrected threshold was *α* = .018. Finally, we added the symptoms’ observed variables in the final model from step 3 to relate the factors with the current symptom levels (RQ4). For the in-lab study, the CFA did not reach a good fit, so subsequent steps were only applied to the online-study, see Supplementary Materials for details.

## Results

### Descriptive information

Descriptives of both samples can be found in Table 1. In the in-lab study, 103 patients had bipolar I disorder with or without psychotic features, 5 bipolar II disorder, 11 schizoaffective disorder, 16 schizophrenia, and 6 another non-affective psychotic disorder. Among relatives and controls, the most common psychopathology was (past) MDD (*n* = 14 and *n* = 15, respectively), but the majority had no history of psychopathology (*n* = 30 and *n* = 48, respectively). In the online-study, participants were not interviewed; based on information from the cohorts we recruited from, 181 patients had BD, and 3 had a psychotic disorder. Two patients from the in-lab study were inpatients, the remainder was outpatient; for the online-study, none of the participants were inpatients. Current depressive symptoms were higher in patients than relatives and controls for both studies (in-lab study: IDS *M*patient = 14.0, *M*relative = 7.5, *M*control = 5.9, *F*(2,249) = 25.8, *p* < .001; online-study: PROMIS *M*patient = 17.8, *M*relative = 12.8, *M*control = 13.9, *F*(2,306) = 20.0, *p* < .001).

**Table 1.** Descriptive information about the samples

	In-lab study (*N* = 272)		Online-study (*N* = 312)	
Group	141 patients (52%)/57 relatives (21%)/74 controls (27%)		183 patients (59%)/79 relatives (25%)/50 controls (16%)	
Sex	138 female (51%)/134 male (49%)		201 female (64%)/111 male (36%)	
Age	52.68 (12.06)		56.61 (12.30)	
Years of education	14.49 (2.37)		14.60 (2.15)	
MID accuracy difference *reward-neutral*	−0.06 (18.57)		4.72 (15.50)	
MID reaction time difference *reward-neutral* (ms)	−5.38 (14.21)		−2.42 (19.25)	
EEfRT percentage hard task choices	35.12 (21.96)		29.84 (22.74)	
EEfRT slope hard task choices at 50% probability	0.10 (0.59)		0.16 (0.80)	
EEfRT slope hard task choices at 88% probability	0.45 (2.00)		0.34 (2.50)	
EEfRT difference in percentage hard task choices between probabilities	15.13 (22.20)		15.83 (23.51)	
Bandit learning rate^a^ coin	0.11 (0.09)		0.11 (0.10)	
Bandit learning rate^a^ social	0.10 (0.09)		0.11 (0.09)	
Bandit total score coin (range: 0–320)	183.48 (12.23)		182.16 (12.15)	
Bandit total score social (range: 0–320)	182.90 (14.25)		183.41 (11.50)	
Bandit win-stay rate^b^ coin	83.10 (14.25)		78.03 (11.02)	
Bandit win-stay rate^b^ social	82.61 (16.13)		76.90 (13.20)	
Bandit lose-shift rate^c^ coin	56.46 (21.15)		53.63 (18.23)	
Bandit lose-shift rate^c^ social	56.87 (22.01)		56.31 (18.64)	
Depression symptoms (IDS in in-lab study, range 0–90; PROMIS in online-study, range 8–40)	10.38 (9.08)		15.91 (6.92)	
Mania symptoms ASRM (patients only, range 0–20)	1.79 (2.39)		1.70 (2.33)	
Positive psychosis symptoms (patients only; PANSS in in-lab study, range 7–49; BASIS–24 in online-study, range 0–16) (mean [sd] [% of participants scoring >3 on at least one psychotic symptom of the PANSS or > 2 on at least one item of the BASIS–24)	9.44 (3.35) (43.0%)		0.25 (0.49) (27.3%)	
Negative symptoms PANSS (patients only, range 7–49)	9.95 (2.92)		NA	
Depressive episodes (self-reported, patients only)	1	8 (6%)	1	20 (11%)
	2	12 (9%)	2	17 (9%)
	3	10 (7%)	3	19 (10%)
	4	9 (6%)	4	11 (6%)
	5+	62 (44%)	5+	36 (20%)
(Hypo)manic episodes (self-reported, patients only)	1	17 (12%)	1	24 (13%)
	2	17 (12%)	2	19 (10%)
	3	15 (11%)	3	11 (6%)
	4	13 (9%)	4	11 (6%)
	5+	52 (37%)	5+	27 (15%)
Psychotic episodes (self-reported, patients only)	1	NA	1	22 (12%)
	2	NA	2	11 (6%)
	3	NA	3	6 (3%)
	4	NA	4	1 (1%)
	5+	NA	5+	2 (1%)

*Note:* Bandit:

a Learning rate: the linear slope of the likelihood of choosing the best arm as a factor of the trial number. This is calculated for the first half of all rounds as most learning takes place then and the slope is likely to get nonlinear in the second half of the runs. The best arm is the one that has the highest reward rate in that round.

b Win-stay rate: the percentage of times a participant chooses the same arm after being rewarded by that arm in the previous trial, averaged across all rounds.

c Lose-shift rate: the percentage of times a participant chooses a different arm after not being rewarded by an arm in the previous trial, averaged across all rounds.

### Covariance between positive valence constructs (RQ1)

**Figure 1.**
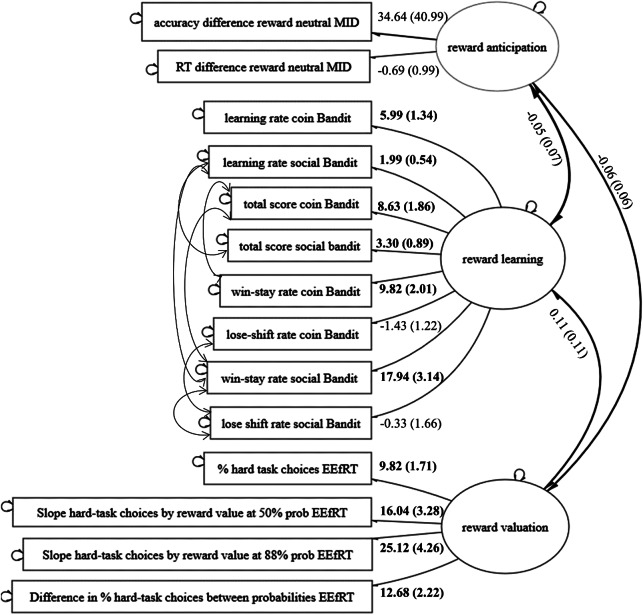
Final model for the online-study with factor loadings and covariances (significant parameters in bold).

The final model for the online-study contained no covariates and several correlated errors between the observed variables in the reward learning factor (see Figure 1). This model showed adequate fit (root mean square error of approximation [RMSEA] = 0.02, standardized root mean square residual [SRMR] = 0.06, Comparative Fit Index [CFI] = 0.98, Tucker–Lewis Index [TLI] = 0.98). Paths from observed task variables to their factor were kept identical to the original plan. The multilevel structure with correction for family relations was maintained, although the fit was similar without this multilevel structure. The three reward-processing factors did not covary (anticipation-effort: *b* = −0.06, standard error [SE] = 0.06, *z* = −0.99, *p* = .32; anticipation-learning: *b* = −0.05, SE = 0.07, *z* = −0.72, *p* = .47; effort-learning: *b* = −0.11, SE = 0.11, *z* = 1.04, *p* = .30).

As mentioned in the methods, the final model for the in-lab study did not meet our fit criteria (RMSEA = 0.07, SRMR = 0.07, CFI = 0.84, TLI = 0.79). Therefore, we base the covariance between factors on the EFA results. Correlations between factors were weak (anticipation-effort: *r* = .14; anticipation-learning: *r* = −.03; effort-learning: *r* = .15).

### Difference in covariance between groups (RQ2)

For the online-study, the multigroup model had a worse fit than the model combining groups (all groups combined Akaike’s Information Criterion (AIC) = 23,233, BIC = 23,410, *χ*^2^ (68) = 76.47; split by group AIC = 23,298, Bayesian Information Criterion (BIC) = 23,826, *χ*^2^ (204) = 292.91, RMSEA = 0.05; *p* < .001). Therefore, we prefer the model combining all groups, that is, the model assuming equal covariance across groups.

### Difference between groups in performance on positive valence constructs (RQ3)

There were no differences between patients, relatives, and controls on any of the indices from the three reward tasks in the in-lab study. Table 2 shows the means and standard deviations (before transformation and outlier winsorization) and model results (after transformation and outlier winsorization) for each outcome. In the online-study, there were several group differences on the EEfRT and Bandit outcomes, but these did not survive multiple comparisons correction (see Table 3). We also examined group differences in scores extracted from the three factors in the final model from the online-study. There were no significant differences (see Table 3). Across the task outcomes, little variance was explained by the family ID random intercept. For several outcomes, the mixed models did not converge, or the random factor accounted for negligible variance, leading us to switch to ANCOVA analyses; details about this are explained in the notes of Tables 2 and 3.

**Table 2.** Group differences in performance on the task outcomes for the in-lab study

	Patients (P)	Relatives (R)	Controls (C)	Group comparison	*b*	SE	df	*t*	*p*	Hedge’s *g*
MID accuracy difference reward-neutral	−0.03 (19.6)	1.54 (17.3)	−1.37 (17.6)	P vs. C	1.57	2.80	240	0.56	.58	−0.05
				R vs. C	3.27	3.31	240	0.99	.32	−0.17
MID reaction time difference reward-neutral (ms)	−5.85 (15.7)	−3.42 (12.7)	−6.06 (12.4)	P vs. C	−0.10	2.18	237.8	−0.05	.96	−0.02
				R vs. C	3.15	2.57	236.3	1.23	.22	−0.21
EEfRT percentage hard task choices	32.5 (23.6)	39.6 (22.9)	36.5 (17.1)	P vs. C	−2.57	3.22	238.3	−0.80	.43	0.19
				R vs. C	6.59	3.81	240.7	1.73	.08	−0.16
EEfRT slope hard task choices at 50% probability	0.07 (0.43)	0.15 (1.02)	0.12 (0.38)	P vs. C	−0.11	0.06	240.8	−1.81	.07	0.30
				R vs. C	−0.005	0.07	237.7	−0.07	.95	0.16
EEfRT slope hard task choices at 88% probability	0.57 (2.30)	0.12 (0.51)	0.49 (2.10)	P vs. C	−0.03	0.07	241	−0.47	.64	0.16
				R vs. C	−0.02	0.08	241	−0.24	.81	0.15
EEfRT difference in percentage hard task choices between probabilities	13.0 (21.1)	17.3 (24.1)	17.4 (22.6)	P vs. C	−2.85	3.21	240.4	−0.89	.38	0.21
				R vs. C	2.85	3.81	239.7	0.75	.46	0.01
Bandit learning rate coin	0.11 (0.09)	0.10 (0.08)	0.12 (0.08)	P vs. C	−0.01	0.01	242	−0.53	.60	0.13
				R vs. C	−0.01	0.02	242	−0.86	.39	0.18
Bandit learning rate social	0.10 (0.09)	0.09 (0.09)	0.10 (0.09)	P vs. C	0.002	0.01	242.3	0.15	.89	0.05
				R vs. C	−0.01	0.02	241.7	−0.65	.52	0.16
Bandit total score coin	183 (12.4)	184 (12.4)	185 (11.9)	P vs. C	−0.88	1.84	239.4	−0.48	.63	0.16
				R vs. C	0.70	2.18	242.0	0.32	.75	0.02
Bandit total score social	183 (11.1)	182 (13.6)	184 (9.2)	P vs. C	−0.23	1.67	242.5	−0.14	.89	0.07
				R vs. C	−1.58	2.00	242.4	−0.79	.43	0.15
Bandit win-stay rate coin	82.3 (14.5)	84.1 (13.5)	83.8 (14.4)	P vs. C	−2.41	3.28	241.6	−0.74	.46	0.13
				R vs. C	−1.86	3.89	240.9	−0.48	.63	−0.01
Bandit win-stay rate social	82.6 (16.5)	79.4 (19.5)	85.1 (11.9)	P vs. C	−1.99	3.47	241.1	−0.57	.57	0.12
				R vs. C	−9.02	4.13	242.7	−2.18	**.03**	0.31
Bandit lose-shift rate coin	54.2 (21.1)	58.3 (20.7)	59.1 (21.5)	P vs. C	−4.30	3.20	241.1	−1.35	.18	0.23
				R vs. C	0.63	3.80	241.2	0.17	.87	0.04
Bandit lose-shift rate social	54.6 (21.7)	61.2 (22.3)	57.9 (22.0)	P vs. C	−3.56	3.32	242.3	−1.07	.28	0.15
				R vs. C	4.77	3.96	242.2	1.21	.23	−0.15

*Note:* Values under ‘patients’, ‘relatives’, and ‘controls’ are means and standard deviations of each group. The top row for each outcome represents the patients–controls comparison, the bottom row represents the relatives–controls comparison. Bold values are significant before multiple comparisons correction, no results were significant after correction. Results for ‘MID accuracy difference reward-neutral’, ‘EEfRT slope hard task choices at 88% probability’, and ‘Bandit learning rate coin’ were based on ANCOVA because no variance was explained by the random intercept.

**Table 3.** Group differences in performance on the task outcomes for the online-study

	Patients (P)	Relatives (R)	Controls (C)	Group comparison	*b*	SE	*t*	*p*	Hedge’s *g*
MID accuracy difference reward-neutral	4.62 (15.0)	3.45 (16.8)	6.98 (14.9)	P vs. C	−4.35	2.92	−1.49	.14	0.18
				R vs. C	−4.78	3.24	−1.48	.14	0.22
MID reaction time difference reward - neutral (ms)	−0.74 (19.8)	−3.75 (19.5)	−5.31 (17.1)	P vs. C	4.31	3.44	1.25	.21	−0.25
				R vs. C	1.89	3.81	0.50	.62	−0.04
EEfRT percentage hard task choices	28.3 (23.8)	30.0 (21.8)	35.0 (20.4)	P vs. C	−0.63	4.33	−0.15	.88	0.30
				R vs. C	2.52	4.69	0.54	.59	0.22
EEfRT slope hard task choices at 50% probability	0.17 (0.79)	0.11 (0.53)	0.23 (1.18)	P vs. C	0.002	0.08	0.02	.98	0.09
				R vs. C	−0.04	0.09	−0.49	.62	0.21
EEfRT slope hard task choices at 88% probability	0.04 (0.53)	0.12 (0.51)	1.79 (6.09)	P vs. C	−0.15	0.07	−1.99	**.048**	0.52
				R vs. C	−0.16	0.08	−2.04	**.043**	0.53
EEfRT difference in percentage hard task choices between probabilities	12.3 (21.7)	15.4 (21.2)	29.1 (29.3)	P vs. C	−12.83	4.49	−2.86	**.005**	0.69
				R vs. C	−10.28	4.85	−2.12	**.036**	0.53
Bandit learning rate coin	0.10 (0.10)	0.12 (0.10)	0.14 (0.10)	P vs. C	−0.03	0.02	−1.62	.11	0.37
				R vs. C	−0.02	0.02	−0.78	.44	0.19
Bandit learning rate social	0.11 (0.10)	0.12 (0.08)	0.09 (0.09)	P vs. C	0.03	0.02	1.58	.12	−0.21
				R vs. C	0.04	0.02	2.08	**.04**	−0.38
Bandit total score coin	180 (12.0)	184 (11.4)	187 (12.3)	P vs. C	−6.24	2.41	−2.60	**.01**	0.61
				R vs. C	−2.60	2.66	−0.98	.33	0.28
Bandit total score social	182 (12.0)	186 (11.4)	185 (9.0)	P vs. C	−2.23	2.34	−0.96	.34	0.32
				R vs. C	2.00	2.62	0.76	.45	−0.06
Bandit win-stay rate coin	77.3 (11.6)	79.5 (10.8)	77.8 (9.2)	P vs. C	−0.58	3.49	−0.18	.87	−0.01
				R vs. C	3.27	3.76	0.87	.39	−0.22
Bandit win-stay rate social	76.9 (13.0)	77.3 (15.3)	76.1 (10.6)	P vs. C	2.74	3.85	0.71	.48	−0.14
				R vs. C	5.21	4.33	1.20	.23	−0.23
Bandit lose-shift rate coin	52.7 (17.8)	52.9 (19.0)	57.8 (18.4)	P vs. C	−4.69	3.75	−1.25	.21	0.29
				R vs. C	−4.67	4.14	−1.13	.26	0.26
Bandit lose-shift rate social	55.3 (19.4)	54.9 (19.5)	61.5 (13.7)	P vs. C	−7.13	3.82	−1.87	.06	0.34
				R vs. C	−7.99	4.30	−1.86	.07	0.38
Anticipation factor	−0.04 (2.02)	−0.20 (2.47)	0.44 (2.21)	P vs. C	−0.73	0.41	−1.78	.08	0.23
				R vs. C	−0.79	0.46	−1.74	.08	0.27
Effort factor	−0.07 (0.63)	−0.06 (0.78)	0.33 (0.91)	P vs. C	−0.22	0.12	−1.75	.08	0.57
				R vs. C	−0.20	0.14	−1.41	.16	0.47
Learning factor	−0.10 (1.15)	0.07 (1.10)	0.23 (1.23)	P vs. C	−0.30	0.22	−1.38	.17	0.29
				R vs. C	−0.12	0.25	−0.50	.62	0.14

*Note:* Values under ‘patients’, ‘relatives’, and ‘controls’ are means and standard deviations of each group. The top row for each outcome represents the patients (P)–controls (C) comparison, the bottom row represents the relatives (R)–controls (C) comparison. Bold values are significant before multiple comparisons correction; no results were significant after correction. Eta-squared values of at least 0.5 are underlined. Results for ‘EEfRT percentage hard task choices’, ‘EEfRT slope hard task choices at 88% probability’, ‘EEfRT slope hard task choices at 88% probability’, ‘Bandit win-stay rate coin’, and ‘Bandit total score social’ were based on linear mixed models. All other outcomes were analyzed with ANOCVA because no variance was explained by the random intercept.

### Association between symptoms and performance on positive valence constructs (RQ3)

For the in-lab study, we examined symptoms in relation to the individual outcome measures of all tasks (see Table 4). The association between depressive symptoms and percentage hard task choices on the EEfRT was the only significant result before multiple comparisons correction, with 3% variance explained, and no associations were significant after multiple comparisons correction. For the online-study, we added the symptom measures to the final CFA model. The results are summarized in Table 5. For depressive symptoms, the model fit remained good, but the level of symptoms was not associated with any of the factors. For mania symptoms, the model fit remained good, but the level of symptoms was again not associated with any of the factors. For psychotic symptoms, the model became unidentified.

**Table 4.** Associations between symptoms and performance on the task outcomes for the in-lab study

Outcome	Predictor	*b*	SE	df	*t*	*p*	Partial eta^2^
MID accuracy difference reward-neutral	Depressive symptoms	−0.10	0.13	240	−0.74	.46	0.002
	Mania symptoms	0.06	0.77	112	0.08	.94	<0.001
	Psychotic symptoms	0.83	0.60	112	1.37	.17	0.02
	Negative symptoms	0.31	0.63	112	0.49	.63	0.002
MID reaction time difference reward-neutral (ms)	Depressive symptoms	−0.07	0.10	234.0	−0.67	.51	0.002
	Mania symptoms	0.65	0.63	110.0	1.04	.30	0.009
	Psychotic symptoms	−0.42	0.48	108.8	−0.87	.39	0.007
	Negative symptoms	−0.05	0.50	101.5	−0.09	.93	<0.001
EEfRT percentage hard task choices	Depressive symptoms	−0.42	0.15	221.7	−2.72	**.007**	0.03
	Mania symptoms	−1.05	0.88	109.0	−1.19	.23	0.01
	Psychotic symptoms	0.44	0.73	112.2	0.60	.55	0.003
	Negative symptoms	−0.76	0.75	88.3	−1.01	.31	0.01
EEfRT slope hard task choices at 50% probability	Depressive symptoms	−0.34	−.28	237.3	−1.20	.23	0.006
	Mania symptoms	−0.02	0.02	113.0	−1.23	.22	0.01
	Psychotic symptoms	0.004	0.01	111.9	0.29	.77	0.001
	Negative symptoms	−0.01	0.01	104.0	−0.41	.68	0.002
EEfRT slope hard task choices at 88% probability	Depressive symptoms	0.02	0.32	241	0.07	.94	<0.001
	Mania symptoms	−0.04	0.02	113.0	−1.96	.05	0.03
	Psychotic symptoms	0.001	0.02	112.1	0.08	.93	<0.001
	Negative symptoms	−0.02	0.01	17.7	−1.49	.16	0.004
EEfRT difference in percentage hard task choices between probabilities	Depressive symptoms	−0.08	0.15	233.8	−0.55	.58	0.001
	Mania symptoms	0.19	0.77	113	0.25	.80	0.001
	Psychotic symptoms	0.09	0.60	113	0.14	.89	<0.001
	Negative symptoms	0.11	0.64	113	0.17	.87	<0.001
Bandit learning rate coin	Depressive symptoms	0.07	0.06	242	1.03	.30	0.004
	Mania symptoms	−0.01	0.003	113	−1.90	.06	0.03
	Psychotic symptoms	0.004	0.003	113	1.50	.14	0.02
	Negative symptoms	−0.003	0.003	113	−1.15	.25	0.01
Bandit learning rate social	Depressive symptoms	−0.04	0.06	235.0	−0.61	.54	0.002
	Mania symptoms	−0.004	0.004	115	−1.28	.21	0.01
	Psychotic symptoms	<0.001	0.003	115	−0.06	.95	<0.001
	Negative symptoms	−0.003	0.003	90.4	−0.92	.36	0.009
Bandit total score coin	Depressive symptoms	0.03	0.09	219.9	0.29	.77	<0.001
	Mania symptoms	−0.27	0.44	113.0	−0.61	.54	0.003
	Psychotic symptoms	0.57	0.36	113.0	1.56	.12	0.02
	Negative symptoms	0.13	0.38	113	0.33	.74	<0.001
Bandit total score social	Depressive symptoms	−0.03	0.08	237.2	−0.35	.73	0.001
	Mania symptoms	−0.06	0.42	107.0	−0.14	.89	<0.001
	Psychotic symptoms	0.25	0.35	114.2	0.75	.46	0.005
	Negative symptoms	0.24	0.33	42.1	0.72	.48	0.01
Bandit win-stay rate coin	Depressive symptoms	−0.06	0.16	237.6	−0.39	.69	0.001
	Mania symptoms	−0.92	0.83	112.7	−1.10	.27	0.01
	Psychotic symptoms	−0.80	0.68	112.1	−1.17	.24	0.01
	Negative symptoms	0.16	0.69	90.0	0.24	.81	<0.001
Bandit win-stay rate social	Depressive symptoms	−0.17	0.17	226.6	−1.05	.30	0.005
	Mania symptoms	−0.001	0.87	91.78	−0.002	.99	<0.001
	Psychotic symptoms	−1.30	0.72	114.8	−1.80	.07	0.03
	Negative symptoms	−0.30	0.66	8.32	−0.46	.66	0.02
Bandit lose-shift rate coin	Depressive symptoms	−0.14	0.16	235.5	−0.91	.37	0.003
	Mania symptoms	−0.36	0.81	112.9	−0.45	.66	0.002
	Psychotic symptoms	0.15	0.67	112.3	0.23	.82	<0.001
	Negative symptoms	−0.70	0.68	112.0	−1.03	.31	0.009
Bandit lose-shift rate social	Depressive symptoms	−0.02	0.16	236.0	−0.11	.91	<0.001
	Mania symptoms	−0.77	0.85	114.5	−0.90	.37	0.007
	Psychotic symptoms	0.76	0.70	114.3	1.08	.28	0.01
	Negative symptoms	−0.51	0.72	101.9	−0.71	.48	0.005

*Note:* Bold values are significant before multiple comparisons correction, no results were significant after correction. Eta-squared values of at least 0.03 are underlined. Results for ‘MID accuracy difference reward-neutral’ (all symptoms), ‘EEfRT slope hard task choices at 88% probability’ (depressive symptoms), ‘EEfRT difference in percentage hard task choices between probabilities’ (mania, psychotic and negative symptoms), ‘Bandit learning rate coin’ (all symptoms), and ‘Bandit learning rate social’ (mania and psychotic symptoms) were based on ANCOVA because no variance was explained by the random intercept.

**Table 5.** Associations between symptoms and performance on the task factors for the online-study

Model	Outcome	*b*	Standard error	*z*	*p*
Depressive symptoms	Anticipation factor	−0.002	0.003	−0.64	.52
	Effort factor	0.001	0.01	0.10	.92
	Learning factor	−0.01	0.01	−1.04	.30
Mania symptoms	Anticipation factor	0.008	0.008	0.95	.34
	Effort factor	0.03	0.04	0.75	.45
	Learning factor	−0.006	0.03	−0.24	.81
Psychotic symptoms	Anticipation factor	0.002	NA	NA	NA
	Effort factor	−0.20	NA	NA	NA
	Learning factor	−0.02	NA	NA	NA

## Discussion

The PVS divides reward-related processes into three constructs: reward responsiveness, reward learning, and reward valuation. The current study aimed to examine how performance on tasks corresponding to these three constructs covaries; how performance differs between adults with mood-psychosis spectrum disorders, those at familial risk, and controls, and how performance relates to mood and psychotic symptoms. We tested this in an in-lab study and an online-study.

The three reward constructs showed weak (nonsignificant) covariance, and we did not find evidence that this covariance was different for individuals with a mood-psychosis spectrum disorder compared to relatives or controls. The weak associations between the reward constructs are in line with the literature: several studies found no correlation between the reward constructs (Duffy et al., 2024; Nguyen et al., 2022; Sailer et al., 2024), or a weak correlation (Geaney et al., 2015). This might imply that the constructs are more distinct than their clustering under the PVS would suggest. The individual outcome variables did cluster together within their reward construct (with the exception of the MID outcome variables), suggesting they have internal consistency. The weak performance of the original MID outcome variables might reflect that it is harder to capture difference scores reliably, or that the MID works better as a brain activation probe than as a behavioral measure.

In the in-lab study, there were no performance differences between groups on any of the outcome variables of the three tasks. In the online-study, no performance differences between groups survived multiple comparisons correction. Based on effect size, there were medium-sized effects of group on the EEfRT slope by reward value, the EEfRT difference in choices between the probabilities, and the overall effort factor. These indicated that controls take into account the reward value and probability more in their choices to exert effort than patients and relatives. Also, based on effect size, not significance, there was a medium-sized difference between patients and controls in the total score on the Bandit coin, with a lower score for patients. This is in line with previous research (Brambilla et al., 2013; Koch et al., 2010; Waltz et al., 2007; Waltz et al., 2011; Yılmaz et al., 2012). Again, looking at effect sizes, it is partially driven by learning rate (Hedge’s *g* = 0.37), but not win-stay rate (Hedge’s *g* = −0.01), and therefore might reflect difficulty identifying, more so than sticking with, the most rewarding option.

Overall, there were fewer impairments in reward-related performance in patients than expected, although the medium effects described above are all in the expected direction. There are several possible explanations for this. The impairments could be overestimated in the literature, related to small samples and publication bias. Meta-analysis could be helpful in this regard, once enough studies have been done on each reward construct. Alternatively, the current study could have underestimated the impairments due to the relatively high-functioning samples. That is, almost all patients were outpatients, had relatively highly educated for this group, and exhibited low levels of current symptoms (see Table 2). This would suggest impairments might not be universal but driven by those with more severe illness history, in a current episode, or with lower education. There is some evidence of effort valuation depending on the current state in BD (Yang et al., 2021). Third, impairments might depend on the task and could be influenced by the cognitive demands of the task. For example, the Bandit task used in this study requires little working memory because the reward history of each arm stays on screen, in contrast to tasks in other studies, like the Iowa Gambling Task or probabilistic versions of the Go/No-go. Working memory impairments are well-known in mood-psychosis spectrum disorders (Bora & Pantelis, 2015) and could affect group differences. For reward tasks that require working memory or other specific cognitive skills, it would be important for future research to test potential confounding by these skills, not just by general cognition or IQ.

For the comparison of participants at familial risk with controls, the literature was inconsistent and partly null (Linke et al., 2020; Hanssen et al., 2020; Mucci et al., 2015; Millman et al., 2020), and therefore our findings are largely consistent with the literature. In addition, the variance in the task outcomes explained by family ID was generally low. Thus, we found no evidence that performance on any of the three PVS constructs could constitute an endophenotype of mood-psychosis spectrum disorders.

Before multiple comparisons correction and when looking at effect sizes, there were more group differences in the online-study than in the in-lab study, particularly in the EEfRT/reward valuation construct. Yet, findings are highly similar after correction. We previously found that participants in the online-study and in-lab study behaved similarly on the EEfRT (Broer et al., 2025, preprint). Note that it was not our intention to directly compare the group differences or associations with symptoms between the settings. The use of the two samples can be seen as a form of replication.

No associations between symptoms and performance on the PVS constructs were significant after multiple comparisons correction. Based on the effect size, 3% of the variance in effort for reward was explained by depressive symptoms, with participants with more current depressive symptoms being less willing to exert effort. This is consistent with the literature (Halahakoon et al., 2020). The same explanations described above for the (lack of) group differences would also apply to these findings on symptom associations.

### Strengths and limitations

Strengths include the use of two samples: (i) a built-in replication, with decent sample sizes; and (ii) the use of standardized, commonly used reward tasks that do not require high (working) memory demands. Yet, the current findings have to be considered in light of several limitations. First, the recruitment strategy and convenience sampling led to selection bias; the samples were relatively well-educated, and the patients were often high-functioning/low in current symptoms. Partially, this is difficult to avoid, as an acute psychotic or manic episode would interfere with participants’ ability to provide informed consent, and those with strong anhedonic symptoms might not be motivated to take part in research. The sampling could have led to an underestimation of group differences and limited variance in the symptom measures. Also, we did not distinguish between diagnostic categories within the group with mood-psychosis spectrum disorders. This was by design as the studies took a dimensional approach, focusing on symptoms that occur across the diagnostic categories (e.g. many of the participants with BD have experienced psychotic episodes). Next, in the online-study, different instruments were used to assess symptom severity for psychosis and depression than in the in-lab study, while negative symptoms were not assessed. It was obvious that we had to find an alternative to the PANSS interview, which was the BASYS24. Also, we aimed to reduce the time burden during the online-study by replacing the IDS with the PROMIS. In addition, we were interested in mechanisms in which familial risk expresses itself, and familial risk occurs heterotypically, that is, across diagnostic boundaries. Yet, it means that we cannot say if patients in certain diagnostic categories showed a different pattern of reward-related behavior than patients in other diagnostic categories. Further, we used one task per reward construct, except for reward learning. The factor analysis would be able to give better evidence of the three-factor structure if we had multiple tasks for each reward construct.

## Conclusion

This study aimed to examine how performance on tasks of reward responsiveness, reward learning, and reward valuation covaries; how performance differs between adults with mood-psychosis spectrum disorders, those at familial risk, and controls; and how performance relates to mood and psychotic symptoms. We examined this in an in-lab setting and an online study. The three reward constructs showed weak (nonsignificant) covariance in all groups. There were a few impairments in reward-related performance in participants with a mood-psychosis spectrum disorder, none of which survived multiple comparisons correction. The comparison of participants at familial risk with controls showed no evidence that performance on any of the three PVS constructs could constitute an endophenotype of mood-psychosis spectrum disorders, though this does not mean they are not endophenotypes, just that the current results do not find support for it. There were no associations between symptoms and performance on the PVS constructs after multiple comparisons correction. We recommend future research examining the contribution of specific cognitive skills to reward-related behavior, and on sources of clinical heterogeneity in reward functioning within the patient group, such as disease severity.

## Supporting information

10.1017/S0033291726103444.sm001 Van Der Weijden-Germann et al. supplementary material Van Der Weijden-Germann et al. supplementary material

## Supplementary material

The supplementary material for this article can be found at http://doi.org/10.1017/S0033291726103444.
