# Supplementary material for: Unraveling reward processing in schizophrenia and bipolar disorder: a multilevel examination of the positive valence system
Source: Psychol Med. 2026 Apr 27;56:e112. doi: 10.1017/S0033291726103444 (PMC13125932; doi:10.1017/S0033291726103444)
Supplement: Van Der Weijden-Germann et al. supplementary material [file S0033291726103444sup001.pdf]

Supplementary Materials

Supplemental Methods

The Monetary Incentive Delay (MID) task

For this task, each trial begins with a cue displayed for 500 milliseconds. This cue indicates whether the participant has the chance to gain or lose points (a circle represents a "win" trial, a square represents a "loss" trial), or if no points are involved (a triangle indicates a "neutral" trial). For win and lose trials, a single horizontal line within the circle or square indicates 75 points, while two horizontal lines indicate 300 points. After the cue, a fixation cross appears for a variable duration (6 seconds minus the durations of the cue and target stimulus). Next, participants must respond as quickly as possible to a target stimulus, a black star, which is displayed on the screen. They press a button box using their dominant hand. Feedback is then provided for 1500 milliseconds, showing whether their response was correct or incorrect (in green or red text, respectively) and the result of the trial: points earned (+75/+300), points lost (-75/-300), or no change (+0). The feedback also displays their cumulative score at that point in the task. Participants need to respond to the target stimulus within a specific time limit, which is personalized for each individual. This "target duration" is determined based on the average of the 16th and 17th slowest reaction times during 24 baseline trials conducted before the main task. The time limit is adjusted after each trial: if the participant's correct response rate is below 66%, 20 milliseconds are added; if it is 66% or higher, 10 milliseconds are subtracted, provided the time limit remains between 150 and 400 milliseconds. The task includes 60 trials, with 12 trials for each condition. Reward conditions have consistently shown to increase accuracy and decrease RT (Barendse, et al., 2024), but the reliability of behavioral measures from the MID is unknown.

Participants of the in-lab study also completed a scanner version of the MID task, with the order of the two versions randomized across participants. Here, we used the out-of-scanner version. To ensure engagement, participants with a non-response rate of 30% or higher were excluded from the analysis.

The Effort Expenditure for Reward Task (EEfRT)

In this task, participants decide between an "easy" task that offers a smaller reward (100 points) or a "hard" task with a larger reward (ranging from 125 to 400 points) during each trial. The easy task requires 30 button presses within 7 seconds using the dominant hand’s index finger, while the hard task requires 100 button presses within 21 seconds using the non-dominant hand’s pinky finger. Before each trial, the probability of receiving the reward (either 50% or 88%) is shown, along with the reward value for the hard

task. Each trial begins with a 1-second fixation period, followed by a 5-second window for participants to choose between the easy or hard task. During this decision period, details about the reward probability and hard task reward magnitude are displayed. If the participant does not decide within 5 seconds, the computer randomly assigns the easy or hard task. After this choice period, the word "Ready" is shown for 1 second, signaling the start of the task. Participants then perform their chosen task, and upon completion, feedback is provided on whether they successfully finished within the time limit and whether they received a reward. The goal of the EEfRT is to accumulate as many points as possible within a 15-minute timeframe. The EEfRT has shown good split-half reliability and validity (Ohmann, et al., 2022).

Trials where the participant did not choose between the two tasks in time (i.e., forced trials), were discarded. EEfRT data from a participant was only analyzed if there were at least 20 non-forced trials.

The Bandit task

The three-armed coin Bandit task consists of 20 rounds, each with 16 trials, and is designed to investigate core processes of reward learning and decision-making. During each round, participants see 16 tokens displayed horizontally at the top of the screen. Their task is to assign each token to one of three arms by pressing the keys 1, 2, or 3 on the keyboard. After assigning a token, feedback is given: a green token indicates earning 1 coin, while a red token means no points were earned. The reward rates for each arm are independent and randomly determined at the start of each round using a beta distribution ($\alpha = \beta = 2$). The accumulated green and red tokens under each arm remain visible throughout the round. Participants have 20 seconds to assign each token. If they fail to respond within this timeframe, the system prompts with "*Are you still there?*" Responding to this question restarts the current round, while no response ends the entire task. Participants are instructed beforehand to identify the arm with the highest reward rate in each round and strive to maximize their total points across all rounds. Reliability of various versions of the Bandit task have been reported as fair-to-good (Mkrtchian, et al., 2023; Schaaf, et al., 2023), although test-retest reliability may be reduced by influences of mood on performance (Schaaf, et al., 2023).

The three-armed social Bandit task mirrors the structure of the three-armed coin Bandit task but replaces coins with compliments as rewards. It is designed to investigate social reward learning. Each round involves three virtual participants, who are fictional but presented as real. These virtual players are depicted with a photograph taken from a public database, along with an initial and age. Before starting the task, all participants complete a 10-item personality questionnaire. They are told that their responses were evaluated by up to sixty virtual players, who could choose to give a compliment (e.g., "*You are honest*") based on the participant's answers or opt to pass if they preferred not to give a compliment. In reality, the personality

questionnaire does not affect the compliments, and participants are informed of this deception during the debriefing. The goal of the task is to maximize the number of compliments received across all rounds.

Bandit data from a participant was only analyzed if there were at least 16 completed rounds (decided separately for the coin and social version). Incomplete rounds were discarded.

Detailed analysis steps

The following steps were employed:

1. Perform assumption checks:
    a. Normality: check plots and skewness and kurtosis. Non-normal variables were transformed with a square-root or cubic transform, depending on their distribution.
    b. Outliers were defined as >3SD away from the mean across the whole sample. We did not find impossible values or cases where a data reporting/entry error has occurred. Thus, outliers were winsorized to 0.1 SD above the highest or below the lowest non-outlier value. In case of non-normality, the interquartile range (IQR) was used instead of the SD and the median was used instead of the mean.
    c. Multicollinearity: we made a correlation matrix of all outcome variables from the three tasks. No correlations were $r \geq .90$ or $r \leq -.90$, so we did not need to remove variables or combine them.
    d. Homoscedasticity: the Bartlett's test was used for normally distributed variables. And Levene's test for non-normally distributed variables. In case of violation of this assumption, standard errors were corrected.
2. Use linear mixed effect modeling to test group differences in all observed task outcome variables (as defined in section 3.3 and listed on the left in Supplemental Figure S1), corrected for age, sex and years of education. Groups are: patients, first-degree relatives, and controls. The only random effect was a random intercept by family ID. This is the first part of the answer to RQ3.
    a. Multiple comparison corrections were applied: a Bonferroni correction is applied adjusted for the average absolute correlation between the outcomes ($r=0.13$) using the D/AP procedure. The threshold after correction was $\alpha=.005$.
    b. Hedge's g effect sizes are reported (without correction for covariates).
    c. In cases where linear mixed effect models did not converge and/or no variation is explained by the random factor, we switched to ANCOVA.
3. Set up the model from Supplemental Figure S1 with the three covariates (but without the 'symptoms' variable) and check model fit. The covariances between the factors answer RQ1.

   a. We set up a multilevel CFA with random intercepts to account for family relationships. We are only interested in the regressions and covariations at the individual level. In case the model does not fit because of no or very little variance at the family level, we remove the multilevel structure.
   b. We used full information maximum likelihood to deal with missing data.
   c. We used a combination of absolute and relative model fit criteria. We considered the model to be of good enough fit if it meets at least three of the four following criteria: RMSEA ≤0.08, SRMR ≤0.08, CFI ≥0.95, TLI ≥0.95.
4. To improve model fit, we tried:
   a. Correlating errors of observed variables within factors
   b. Removing observed task variables with a low loading on their factor
   c. Removing paths from covariates to factors in case of low loadings
   d. Seeing if any observed task variables load on a second factor. If so, allow this cross-loading.

   For the in-lab study, the steps under point 4 did not lead to a good fitting model. Thus, we ran an exploratory factor analysis (not preregistered) with three factors and promax rotation. This showed low loadings for the MID variables on all factors (see Supplementary Table S1). We replaced the MID outcome variables with total money won and percentage correct for large reward trials. This led to a structure that aligned with the three factors as defined in the CFA (see Supplementary Table S2). We refitted this model as a CFA. This model still did not meet our model fit criteria. Therefore, steps 5-7 were only applied to the online-study. For the in-lab study, we compared the individual task outcome measures between groups and related them to symptom levels (following the same procedures and thresholding as for the linear mixed models in step 2).
5. Once we had a good fitting model across the whole dataset, we used multigroup CFA to compare the factor covariances between groups (RQ2). A model with factor covariances restricted to be equal across groups was compared to a model with factors covariances allowed to vary, by using a chi-square test. With this type of analysis, the change in fit after versus before separating the groups provides information about whether the groups differ in covariation. That is, if the model with groups separated would fit better, we would conclude that there is a difference in covariation, since the model with groups separated allows this covariation to be estimated differently for each group.
6. Extract factor scores from the three factors using lavPredict and apply ANCOVA to compare these scores between groups (second part of answer to RQ3). We applied multiple comparisons in the same way as in step 2, but in this case correcting across the three factors, and reported Hedge's g effect sizes. The threshold after correction was α=.018.

7. Add the symptoms' observed variable in the final model from step 3-4 to relate the factors with the current symptom levels (RQ4), each tested in a separate model.

Table S1. Factor loadings of the exploratory factor analysis with original variables (loadings above 0.2 or below -0.2 are highlighted)

MID accuracy difference *reward - neutral*	0.11	0.08	0.13
MID reaction time difference *reward - neutral* (ms)	-0.06	0.00	-0.05
EEfRT percentage hard task choices	-0.02	-0.16	**0.51**
EEfRT slope hard task choices at 50% probability	0.04	0.05	**0.36**
EEfRT slope hard task choices at 88% probability	0.00	-0.05	**0.59**
EEfRT difference in percentage hard task choices between probabilities	0.01	-0.05	**0.58**
Bandit learning rate coin	0.19	**0.45**	-0.15
Bandit learning rate social	-0.10	**0.22**	**0.21**
Bandit total score coin	-0.04	**0.44**	0.12
Bandit total score social	-0.13	**0.31**	**0.29**
Bandit win-stay rate coin	-0.04	**0.84**	-0.16
Bandit win-stay rate social	**-0.26**	**0.57**	-0.10
Bandit lose-shift rate coin	**0.62**	-0.11	0.04
Bandit lose-shift rate social	**1.03**	0.08	-0.08

Table S2. Factor loadings of the exploratory factor analysis with new outcome variables for the MID (loadings above 0.2 or below -0.2 are highlighted)

MID accuracy difference reward - neutral	-0.01	**0.75**	0.01
MID reaction time difference reward - neutral (ms)	0.05	**0.90**	-0.03
EEfRT percentage hard task choices	-0.04	0.05	**0.39**
EEfRT slope hard task choices at 50% probability	0.07	-0.11	**0.41**
EEfRT slope hard task choices at 88% probability	0.07	0.00	**0.52**
EEfRT difference in percentage hard task choices between probabilities	0.05	0.06	**0.51**
Bandit learning rate coin	**0.21**	-0.04	0.10
Bandit learning rate social	**0.32**	0.07	0.15
Bandit total score coin	**0.45**	0.02	0.17
Bandit total score social	**0.43**	0.00	**0.22**
Bandit win-stay rate coin	**0.67**	0.01	0.00
Bandit win-stay rate social	**0.67**	0.00	-0.15
Bandit lose-shift rate coin	**-0.54**	0.03	**0.41**
Bandit lose-shift rate social	**-0.59**	-0.01	**0.49**

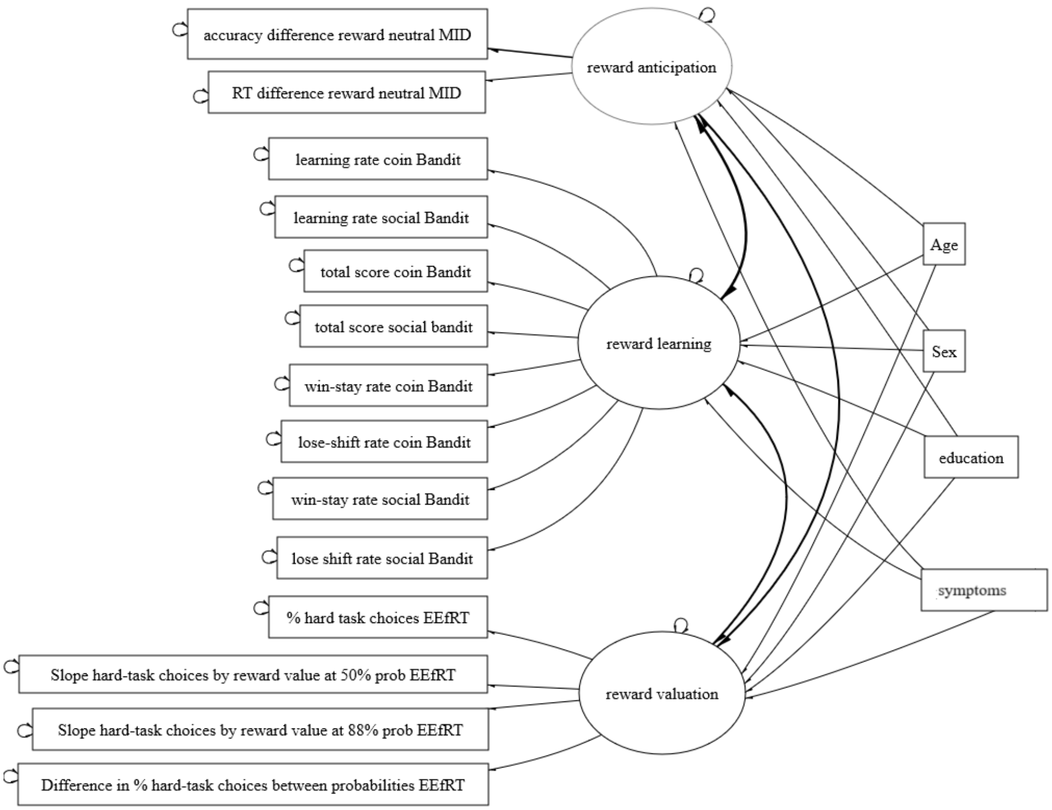

Figure S1. Model set up to test covariance between positive valence constructs
